# Human immunodeficiency virus‐associated pulmonary sarcoidosis in a Japanese man as a manifestation of immune reconstitution inflammatory syndrome

**DOI:** 10.1002/ccr3.3438

**Published:** 2020-10-27

**Authors:** Hideta Nakamura, Masao Tateyama, Daisuke Tasato, Shusaku Haranaga, Futoshi Higa, Akiko Matsuzaki, Naoki Yoshimi, Jiro Fujita

**Affiliations:** ^1^ Department of Infectious, Respiratory, and Digestive Medicine University of the Ryukyus Graduate of School of Medicine Nishihara Japan; ^2^ Department of Respiratory Medicine Hokubu Chiku Ishikai Hospital Nago Japan; ^3^ Department of Respiratory Medicine National Hospital Organization Okinawa National Hospital Ginowan Japan; ^4^ Department of Pathology Urasoe General Hospital Urasoe Japan; ^5^ Department of Pathology Okinawa Red Cross Hospital Naha Japan

**Keywords:** antiretroviral therapy, human immunodeficiency virus infection, immune reconstitution inflammatory syndrome, sarcoidosis

## Abstract

Asymptomatic pulmonary sarcoidosis can develop after starting antiretroviral therapy. The decision on whether to treat sarcoidosis with corticosteroids should be based on the disease severity.

## Graphical abstract

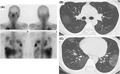

## 1 INTRODUCTION

A 26‐year‐old HIV‐positive hemophiliac man was hospitalized with chronic renal failure. He had been taking antiretroviral therapy for 3.5 years. While investigating his renal function, Gallium‐67 scintigraphy detected bilateral segmental lung uptake. Lung biopsy revealed noncaseous granulomas, which stained negative for microorganisms. Hence, he was diagnosed with pulmonary sarcoidosis.

The development of sarcoidosis in patients with human immunodeficiency virus (HIV) had rarely been reported before the antiretroviral therapy (ART) era. The relative deficiency of CD4^+^ T‐lymphocytes in untreated HIV infection is considered to hamper the development of sarcoidosis because CD4 T cells play a central role in granuloma formation. Sarcoidosis has been reported as a manifestation of immune reconstitution inflammatory syndrome (IRIS) in patients with HIV who are receiving ART.^1^, ^2^ We report about a Japanese hemophilic man with HIV infection receiving ART who developed asymptomatic pulmonary sarcoidosis.

## 2 CASE HISTORY AND EXAMINATION

A 26‐year‐old HIV‐infected Japanese hemophiliac man was admitted to our hospital for investigation of his chronic renal failure. He had been infected HIV 21 years previously by a contaminated blood product transfusion. He had initiated ART 9 years previously; discontinued ART due to nonadherence; and re‐initiated ART 3.5 years previously, with a CD4 count of 155 cells/μL and HIV viral load of 100 000 copies/mL at the time of ART initiation. Twelve months after initiating ART, his CD4 count had increased 250 cells/μL, and his viral load was undetectable. In the interim, his renal function gradually worsened despite the use of an angiotensin II receptor blocker.

On admission, he had a blood pressure of 142/72 mm Hg, heart rate of 84/min, and a body temperature of 36.1°C. He had no symptoms of uremia, and his physical examination was unremarkable. His CD4 count was 460 cells/μL, and his HIV viral load was undetectable.

## 3 INVESTIGATIONS AND TREATMENT

**FIGURE 1:**
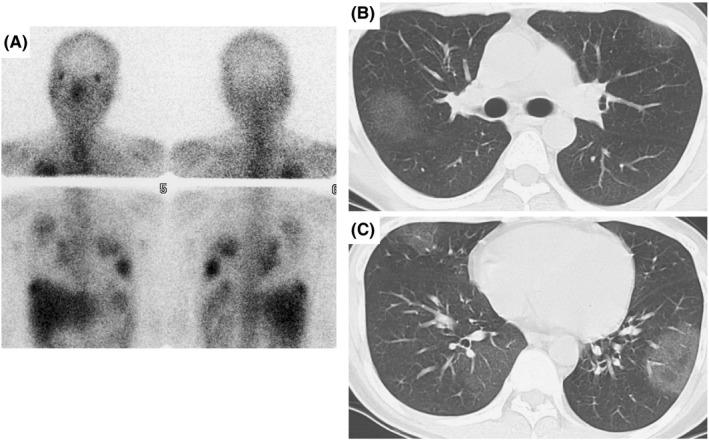
Imaging findings. A, Gallium‐67 scintigraphy showed bilateral segmental lung uptake, and no uptake in kidney; B and C, Chest computed tomography revealed bilateral segmental ground grass opacities

Gallium‐67 (Ga‐67) scintigraphy was performed to evaluate his renal function. Although no uptake was detected in kidney, bilateral segmental lung uptakes were detected as an incidental finding (Figure 1). Chest computed tomography, performed the same day, revealed bilateral ground grass opacities (Figure 1). His tuberculin skin test was negative. Blood tests revealed an increase in the levels of serum blood urea nitrogen and creatinine. All the other blood tests, including the complete blood count, leukocyte differential count, CD4 count, CD4/CD8 ratio, calcium, C‐reactive protein, KL‐6, and liver function tests, were in the normal range (Table 1). The pulmonary function test results were normal. The urinalysis showed mild proteinuria and an elevated β2‐microglobulin level. Bronchoscopy to obtain samples of lung tissue was unsuccessful. Therefore, video‐assisted thoracic surgery was performed along with factor VIII infusion.

**TABLE 1:** Laboratory findings on admission

Complete blood count		Serology	
White blood cells (cells/μL)	9700	C‐reactive protein (mg/dL)	0.94
Neutrophil (%)	62	KL‐6 (U/L)	600
Eosinophil (%)	4	ACE (U/L)	11.5
Lymphocyte (%)	25.3		
Monocyte (%)	8.5	Urinalysis	
Hemoglobin (g/dL)	10.1	Protein	1+
Platelets (×10^4^/μL)	27.1	Glucose	Negative
		Urinary β‐2microglobulin (mg/L)	12.4
HIV‐related variables			
CD4^+^ T‐lymphocyte (cells/μL)	460	Pulmonary function test	
CD8^+^ T‐lymphocyte (cells/μL)	1177	VC (L)	3.83
CD4/CD8 ratio (%)	0.39	%VC (%)	94.2
HIV viral load (copies/mL)	<50	FEV_1.0_ (L)	3.68
		FEV_1.0_% (%)	96.1
Biochemistry			
Blood urea nitrogen (mg/dL)	41		
Creatinine (mg/dL)	3.86		
Aspartate aminotransferase (U/L)	14		
Alanine aminotransferase (U/L)	15		
Calcium (mg/dL)	9.4		
Lactate dehydrogenase (U/L)	199		

Abbreviations: %VC, percent vital capacity; ACE, angiotensin‐converting enzyme; FEV_1.0%_, forced expiratory volume 1.0 s percent; FEV_1.0_, forced expiratory volume in 1 s; VC, vital capacity.

**FIGURE 2:**
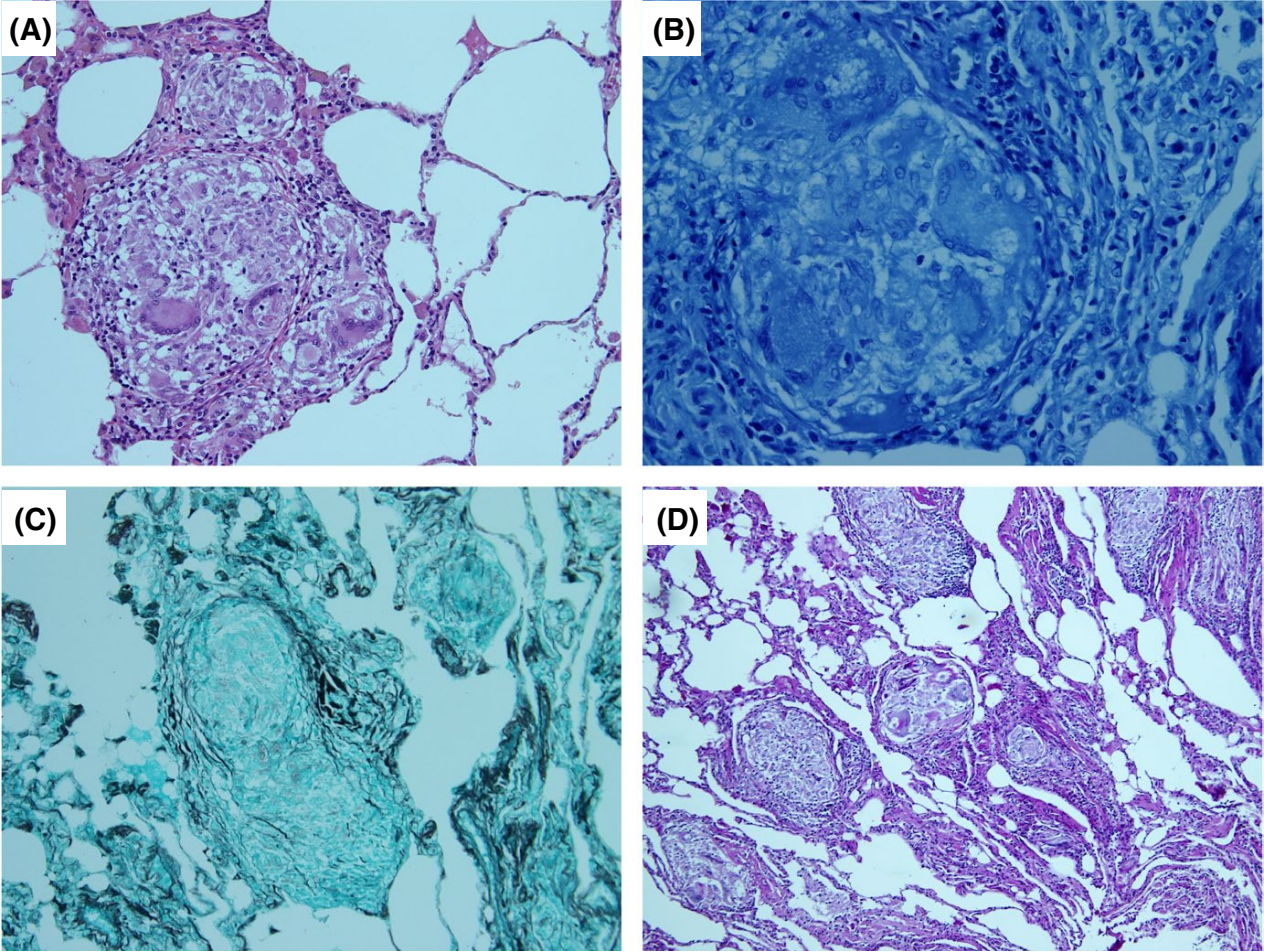
Histopathology of resected lung tissue. A, noncaseous granuloma containing Langerhans giant cells and star bodies (hematoxylin and eosin stain, magnification ×400); B, Ziehl‐Neelsen stain, magnification ×1000; C, Grocott staining, magnification ×100; D,: Periodic acid‐Schiff staining, magnification ×100. The Ziehl‐Neelsen‐, Grocott‐, and periodic acid‐Schiff‐stained specimens were negative for pathogens

Lung histopathology revealed noncaseous granulomas containing Langerhans giant cells and star bodies (Figure 2). No organisms were detected using Ziehl‐Neelsen, Grocott methenamine silver, and periodic acid‐Schiff staining. Based on these findings, pulmonary sarcoidosis was diagnosed. No other organ disease was detected. His chronic renal failure was considered to be unrelated to sarcoidosis, given the lack of Ga‐67 uptake by the kidneys on scintigraphy in the kidney, and was attributed to HIV‐associated nephropathy and/or drug‐induced interstitial nephritis. Because his sarcoidosis was asymptomatic, corticosteroid was not administered.

His pulmonary lesions resolved spontaneously over the course of several months.

## 4 DISCUSSION

Sarcoidosis is a multisystem disease of unknown etiology that is characterized pathologically by noncaseous granulomas with a predilection for the lungs.^3^ CD4^+^ T‐lymphocytes may be important to the pathogenesis of sarcoidosis because many patients with sarcoidosis have CD4^+^ T‐lymphocyte accumulation at the sites of granulomatous inflammation, despite modest peripheral lymphopenia.^4^ In patients with HIV infection, circulating CD4^+^ T‐lymphocytes are progressively destroyed leading to an increased risk of opportunistic infections.^5^ Divergent immunological abnormalities in these two conditions may explain the phenomenon of sarcoidosis‐provoking overactive CD4^+^ T‐lymphocyte activity in the presence of HIV‐induced CD4^+^ T‐lymphocyte depletion, leading to attenuation of granulomata formation.^6^

**FIGURE 3:**
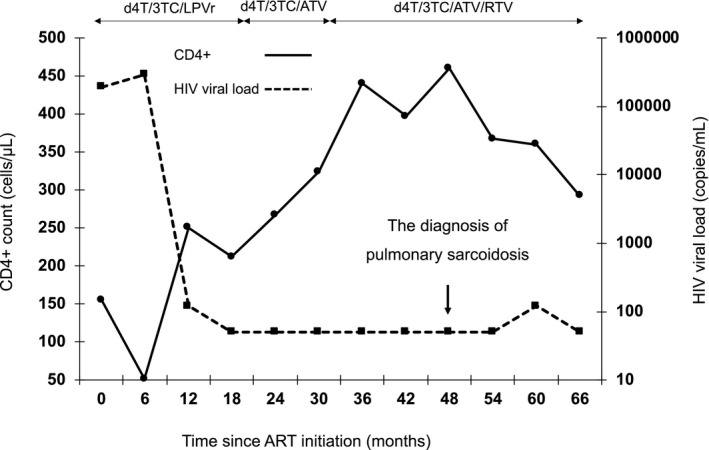
The clinical course. Abbreviations: ATV, atazanavir; d4T, stavudine; LPVr, lopinavir/ritonavir; RTV, ritonavir; 3TC, lamivudine

In HIV patients, sarcoidosis has been described after ART initiation, as a manifestation of IRIS.^1^ The recovery of the immune system can result in an enhanced inflammatory reaction to subclinical autoimmune disease and infection.^7^ A unique feature of sarcoidosis among individuals on ART is that the mean interval between ART initiation and the onset of sarcoidosis is much longer (several months) than that reported for IRIS of infectious origin (a few weeks).^8^ Foulon et al^1^ reported 8 HIV patients with sarcoidosis who had been on ART for a mean of 29 months (range: 3‐43 months) at the time of diagnosis. In our case, the patient developed pulmonary sarcoidosis 3.5 years after ART initiation following the recovery of CD4^+^ T cells (Figure 1). Given the chronological order (Figure 3) and previous studies, the pulmonary sarcoidosis was probably attributable to IRIS. The long interval between initiation of ART and onset of sarcoidosis may be explained by the involvement of a naïve CD4^+^ T cell pool that expands later than the memory T cell pool in HIV‐infected patients receiving ART.^1^

The lack of hilar and mediastinal lymphadenopathy was an unusual finding in this case, since radiographic patterns are similar in HIV negative patients; chest computed tomography findings include thickened interlobular septa, reticular opacities, and ground‐glass opacities.^8^ Serum angiotensin‐converting enzyme and C‐reactive protein levels, which are biomarkers of sarcoidosis, were not increased.^9^ Despite these atypical findings, the diagnosis of sarcoidosis was consistent with the lung histopathology.

Nevertheless, the potential existence of undiagnosed pathogens that can cause granulomatous lesions should be considered. For example, granulomatous *Pneumocystis jirovecci* pneumonia can occur as a manifestation of delayed IRIS.^10^ Molecular analysis of granulomatosis lesions may be useful for determining the antigens that are responsible for granuloma formation.

The characteristics of HIV‐associated sarcoidosis are similar to those of non‐HIV‐related sarcoidosis.^1^ Respiratory and constitutional symptoms occur in approximately 50% of individuals,^1^ but asymptomatic cases of pulmonary sarcoidosis have been reported among HIV‐positive patients.^1^, ^11^ Extra‐thoracic lesions may occur in almost any organ.^12^ Ferrand et al^13^ reported an HIV patient with systemic sarcoidosis, with hypercalcemia and renal failure, occurring as a manifestation of IRIS. Although we could not perform a pathological examination of the kidney in our patient, his renal failure is unlikely to have been caused by sarcoidosis given the negative result of Ga‐67 scintigraphy. The spontaneous resolution of the ground grass opacities in his lungs, despite the progression of renal failure also suggests that his renal failure was unrelated to his sarcoidosis.

The indication for corticosteroid therapy depends on the disease severity. Foulon et al^1^ reported that 5 of 11 patients improved or remained stable without steroid therapy. Our patient's pulmonary lesions resolved spontaneously.

In conclusion, asymptomatic pulmonary sarcoidosis was discovered during the investigation of chronic renal failure in an HIV‐positive hemophiliac patient on ART. Although his clinical presentation was atypical, the pathological examination of lung tissue ruled out other potential causes. The decision to use steroids to treat pulmonary sarcoidosis should be based on the severity of the condition.

## CONFLICT OF INTEREST

The authors declare that there are no conflicts of interest.

## AUTHOR CONTRIBUTIONS

HN: compiled with the draft of the manuscript. MT, DT, SH and FH: interpreted the data and assisted in the preparation of the manuscript. AM and NY: analyzed pathological findings. JF: supervised and approved the manuscript to be published.

## ETHICAL APPROVAL

Ethical approval was not required for this case report. Written informed consent for publication was obtained from the patient.

## Data Availability

The data that support the findings of this study are available from the corresponding author upon reasonable request.
